# A Case of Prolonged Wernicke's Encephalopathy Successfully Treated With Long-Term High-Dose Thiamine

**DOI:** 10.7759/cureus.85163

**Published:** 2025-06-01

**Authors:** Ryo Mizui, Ryohei Takada, Kanehiro Ikuno, Shintaro Araki, Takashi Okada

**Affiliations:** 1 Department of Psychiatry, Nara Medical University, Kashihara-shi, JPN

**Keywords:** alcoholism, long-term high-dose, thiamine, thiamine deficiency, wernicke's encephalopathy

## Abstract

Wernicke's encephalopathy (WE) is a neurological disorder caused by thiamine deficiency. This condition is characterized by impaired consciousness, eye movement disorders, and gait ataxia. Early diagnosis and thiamine treatment are critical owing to the condition's high mortality rate and risk of progression to Korsakoff syndrome. However, WE is often underdiagnosed, and treatment guidelines remain limited. In this case study, we report the case of a patient diagnosed with WE shortly after improvement from alcohol withdrawal delirium. High-dose thiamine administration for approximately nine weeks resulted in a marked improvement in consciousness. This case underscores the importance of considering WE in patients with alcohol use disorder who exhibit episodes of impaired consciousness and poor food intake, specifically when guided by Caine's criteria. It also highlights the potential benefit of high-dose intravenous thiamine administration, particularly in the early stages of treatment, and suggests that prolonged treatment may still yield improvements, even in cases with delayed recovery.

## Introduction

Wernicke's encephalopathy (WE) is a neurological disorder caused by thiamine (vitamin B1) deficiency and is characterized by impaired consciousness, eye movement disorders, and gait ataxia. It frequently occurs in individuals with alcohol dependence and is associated with a mortality rate of 10-20% if left untreated. Furthermore, without appropriate intervention, approximately 80% of patients may develop Korsakoff syndrome [1]. Korsakoff syndrome is characterized by marked impairment in memory formation, disorientation, and confabulation, along with physical signs such as peripheral neuropathy and ataxia. Once the syndrome develops, it is generally considered irreversible, and the associated mortality rate is extremely high [1]. Early diagnosis of WE is therefore critical; however, the clinical prevalence (0.04-0.13%) is markedly lower than that reported in general population autopsy studies (0.8-2.8%), indicating a high rate of underdiagnosis, especially in patients with alcohol use disorder [2]. Timely administration of thiamine is essential for a favorable outcome. Although high-dose intravenous thiamine is considered the standard treatment, the optimal dosage and duration of administration remain unclear owing to insufficient supporting evidence [3]. Current practice relies on recommendations from several international guidelines [4-7], and treatment must be tailored to the individual. Here, we report a case of WE diagnosed shortly after the resolution of alcohol withdrawal delirium. Here, we report a case of WE diagnosed shortly after the resolution of alcohol withdrawal delirium. The patient showed marked clinical improvement after approximately nine weeks of high-dose thiamine treatment.

## Case presentation

A 57-year-old woman began drinking alcohol socially at the age of 20 years. She worked full-time as a nurse, with an eight-hour shift five days a week, occasionally including night shifts, and lived with both parents. During this time, she developed work-related stress and gradually experienced sleep-onset insomnia. To aid sleep, she began consuming approximately 1 L of beer daily. Approximately six years before the index year (X-6), the patient's alcohol intake before bed increased, and she began experiencing appetite loss. In March of that year, the patient presented to our department and was diagnosed with alcohol use disorder. Despite counseling, her alcohol consumption persisted. By four years before the index year (X-4), she had begun drinking in the mornings on her days off, sometimes consuming up to 4 L of a 7% whisky-based beverage. By two years before the index year (X-2), she was diagnosed with alcoholic hepatitis at a workplace health check-up, but did not reduce her alcohol intake at that time. By August of the index year, she drank daily in the morning, stopped eating regular meals, and was on work leave. She experienced general fatigue and stopped drinking on September 6. On September 8, she was brought to our department because of agitation and reports of visual hallucinations of “flying insects” upon waking and was admitted. The patient was restless, disoriented to time and place, and had a Mini-Mental State Examination (MMSE) score of 12. Clinical findings included visual hallucinations, a heart rate of 105 bpm, a temperature of 37.4°C, bilateral hand tremors, and mild diaphoresis. Although overt gait ataxia was not observed, assessment of eye movements was limited because of poor cooperation. Blood tests revealed mild hepatic dysfunction and malnutrition (albumin: 3.5 g/dL; potassium: 3.2 mEq/L) (Table 1).

**Table 1:** Blood test results.

Test item	Result	Normal range	Unit
Albumin	3.5	3.8–5.3	g/dL
Potassium	3.2	3.5–5.0	mEq/L
Vitamin B1 (thiamine)	33.3	24.0–66.0	ng/ml
Vitamin B12	363.1	233.0–914.0	pg/ml

**Figure 1:**
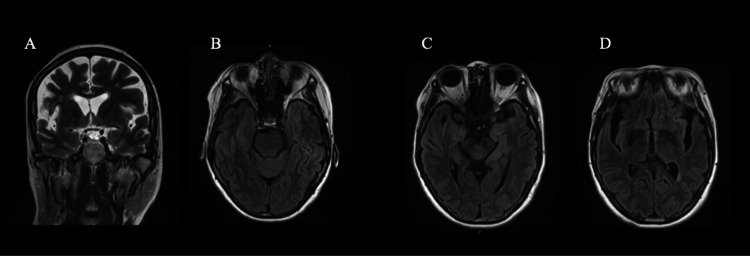
Magnetic resonance images of the patient’s brain. (A) Coronal view (T2-weighted image): mild atrophic changes are observed in both cerebral hemispheres. (B)–(D) Axial views (FLAIR: fluid-attenuated inversion recovery): No apparent abnormal signal was observed in (B) cerebellar vermis, (C) cerebral aqueduct, or (D) medial thalamus and periventricular region of the third ventricle.

On physical examination, the patient had a height of 162 cm, a weight of 41 kg, and a BMI of 15.6, all of which indicated a state of malnutrition. However, blood thiamine levels (revealed five days later) were within the normal range (33.3 ng/mL). Brain magnetic resonance imaging (MRI) showed no abnormalities (Figure 1).

**Figure 2:**
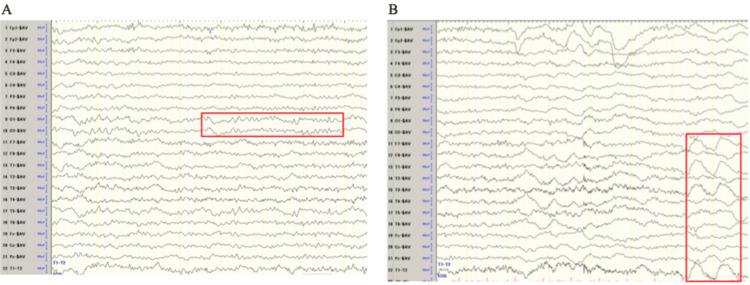
Electroencephalogram (EEG) findings (average reference montage). (A) Baseline activity: an alpha rhythm at 8–9 Hz was observed, predominantly over the posterior regions. (B) Intermittent slow waves were observed predominantly in the bilateral anterior temporal regions.

Electroencephalogram (EEG) revealed a basic rhythm of 8-9 Hz with slow waves (δ and θ) in the bilateral anterior temporal regions (Figure 2).

Although blood vitamin B3 levels were not measured, pellagra was ruled out owing to the absence of characteristic skin and gastrointestinal symptoms.

Two days after alcohol cessation, the patient developed disorientation, autonomic instability, and hallucinations, leading to a diagnosis of alcohol withdrawal delirium. Subsequently, diazepam (15 mg/day) was administered orally. Given her malnutrition and poor oral intake before admission, thiamine deficiency was suspected, and intravenous thiamine therapy (1500 mg/day) was initiated. Hallucinations and autonomic symptoms resolved by day six of hospitalization; however, her consciousness remained impaired (MMSE score: 11), leading to a diagnosis of Wernicke’s encephalopathy, with reference to Caine's criteria. The patient did not display any gait disturbances or nystagmus. From day seven, the dose of diazepam was reduced to 8 mg/day and that of thiamine to 1000 mg/day. Diazepam was discontinued on day 14, whereas thiamine was continued at 500 mg/day until further improvement.

**Figure 3:**
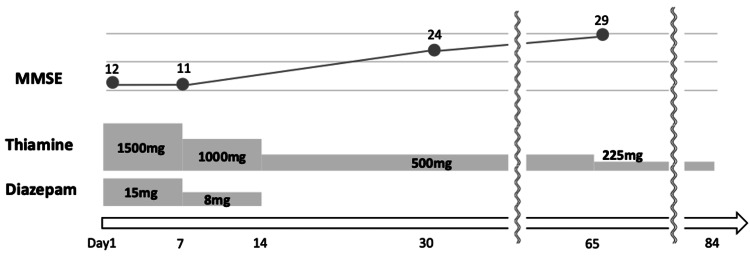
Treatment and clinical course. MMSE: Mini-Mental State Examination.

Although there were no signs of confabulation or Korsakoff syndrome, the patient’s impaired consciousness and behavioral disturbances persisted. The patient could not recall the date or her occupation and sometimes played with food using her hands. Around day 30, the patient’s consciousness markedly improved, and she could correctly state the date and her occupation. Moreover, her MMSE score improved to 24. By day 65, the patient’s MMSE score was 29, which prompted a transition to oral thiamine (225 mg/day). The EEG on day 72 showed no slow waves with a basic rhythm of 10-11 Hz. The patient joined an alcohol rehabilitation program and was discharged on day 84 without relapse. She remained abstinent and returned to work during outpatient follow-up. The detailed clinical course is shown in Figure 3.

Written informed consent was obtained from the patient for publication, and all personal data have been fully anonymized in accordance with journal guidelines.

## Discussion

Given the underdiagnosis of WE, strategies for its early recognition are essential. The classical triad--impaired consciousness, eye movement disorders, and gait ataxia--is present in only a few cases. Sechi et al. [2] reported that 82% of patients with WE exhibited impaired consciousness, whereas eye movement disorders and gait ataxia were observed in 29% and 23%, respectively. Only 16-38% of patients exhibited all three features. Caine et al. [8] proposed a diagnostic criterion requiring any two of the following four features: (1) dietary deficiency, (2) oculomotor abnormalities, (3) cerebellar dysfunction, and (4) altered mental state or memory impairment. This method yields a sensitivity of 85%. Blood thiamine levels are often misleading, as they may appear normal despite metabolic or central nervous system-specific deficiency, and the results are often delayed [9]. MRI may reveal characteristic symmetrical hyperintensities in the medial thalamus, periaqueductal gray, mammillary bodies, or cerebellar vermis on T2-weighted or fluid-attenuated inversion recovery (FLAIR) sequences. However, its diagnostic sensitivity is only 53%, albeit with a high specificity of 93% [10]. In the present case, despite normal thiamine levels and MRI findings, a diagnosis of WE was made based on the Caine criteria (altered mental status and dietary deficiency).

WE may coexist with alcohol withdrawal delirium owing to severe nutritional compromise and accelerated thiamine metabolism. Therefore, early intervention using thiamine therapy is critical. Given its low oral bioavailability (maximum absorption of ~4.5 mg/day) [11], intravenous administration is the standard approach. Although thiamine is well tolerated even at high doses, there is insufficient evidence to define the optimal dosage and duration of administration [3]. Moreover, existing guidelines vary considerably [4-7], with some omitting duration recommendations (Table 2).

**Table 2:** Comparison of guidelines for treatment of Wernicke encephalopathy.

	Thiamine dose, frequency	Route	Duration
The European Academy of Neurology (EAN) [4]	① 500 mg, three times daily	Intravenous	① Three days
	↓		
	② 250 mg/day		② Until symptom resolution
The Royal College of Physicians report [6]	① 500 mg, three times daily	Intravenous	① Three days
	↓		
	② 250 mg/day		② Until symptom resolution
Canadian Research Initiative in Substance Misuse[7]	① 200-300 mg/day	① Parenteral	① At least five days
	↓		
	② 200 mg/day	② Oral	② Unspecified
American Psychiatric Association [5]	50-100 mg, once daily	Oral or intravenous	Unspecified

In this case, thiamine was administered intravenously at a dose of 1500 mg/day for seven days, followed by 1000 mg/day for another seven days, and 500 mg/day for 40 days--a total treatment duration of approximately nine weeks.

Initially, we planned to administer 1500 mg of thiamine for three days in accordance with existing guidelines. However, as no clinical improvement was observed, we decided to continue high-dose therapy until improvement was achieved, based on previous case reports by Carota and Paparrigopoulos. On the other hand, frequent intravenous administration carries the risk of phlebitis and may lead to reduced mobility, placing a burden on the patient. Therefore, we adopted the current regimen. Specifically, on day three, we decided to extend the 1500 mg/day dose through day seven, and on day seven, we determined to administer 1000 mg/day through day 14. This regimen resulted in significant clinical improvement of cognitive function, with the MMSE score increasing from 12 to 29. Further accumulation of case reports is essential to clarify the validity of this treatment approach. The pathophysiology of thiamine-deficiency-related neurotoxicity remains unclear. Neither symptom severity, biomarker changes (e.g., thiamine levels, transketolase activity, and urinary excretion) nor MRI findings can reliably predict recovery [12]. While impaired consciousness in WE typically resolves within days to two weeks, this case required nine weeks for substantial cognitive recovery. Some previous reports have described a similar delayed recovery. Carota et al. [12] reported a patient with WE secondary to alcoholism who recovered after two months of enteral thiamine therapy (600 mg/day), following a four-month untreated period. Paparrigopoulos et al. [13] described another patient with persistent cognitive symptoms despite one month of intramuscular thiamine administration at 100 mg/day, which resolved after an increase to 900 mg/day (600 mg orally + 300 mg intravenously) for three weeks. These findings suggest that delayed or insufficient initial therapy contributes to prolonged symptoms.

## Conclusions

Here, we report a case of Wernicke’s encephalopathy that was diagnosed early despite a normal blood thiamine level, and in which delayed recovery was successfully achieved through extended high-dose thiamine therapy. The pathophysiology and prognosis of WE remain poorly understood, and early diagnosis is often challenging. Vigilance is crucial, especially when treating patients with alcohol use disorder, and clinicians should consider using diagnostic criteria. Furthermore, aggressive and prolonged thiamine therapy may be warranted, even in delayed or clinically ambiguous cases. Further case reports are necessary to improve diagnostic accuracy, define optimal treatment protocols, and identify factors contributing to prolonged recovery.
